# A method for ordinal outcomes: The ordered stereotype model

**DOI:** 10.1002/mpr.1801

**Published:** 2019-09-30

**Authors:** Daniel Fernandez, Ivy Liu, Roy Costilla

**Affiliations:** ^1^ Research and Development Unit, Parc Sanitari Sant Joan de Déu Fundació Sant Joan de Déu, CIBERSAM Barcelona Spain; ^2^ School of Mathematics and Statistics Victoria University of Wellington Wellington New Zealand; ^3^ Queensland Alliance for Agriculture and Food Innovation (QAAFI) University of Queensland Brisbane Australia

**Keywords:** goodness–of–fit, ordered stereotype model, ordinal data, proportional odds model

## Abstract

**Objective**: The collection and use of ordinal variables are common in many psychological and psychiatric studies. Although the models for continuous variables have similarities to those for ordinal variables, there are advantages when a model developed for modeling ordinal data is used such as avoiding “floor” and “ceiling” effects and avoiding to assign scores, as it happens in continuous models, which can produce results sensitive to the score assigned. This paper introduces and focuses on the application of the ordered stereotype model, which was developed for modeling ordinal outcomes and is not so popular as other models such as linear regression and proportional odds models. This paper aims to compare the performance of the ordered stereotype model with other more commonly used models among researchers and practitioners.

**Methods**: This article compares the performance of the stereotype model against the proportional odd and linear regression models, with three, four, and five levels of ordinal categories and sample sizes 100, 500, and 1000. This paper also discusses the problem of treating ordinal responses as continuous using a simulation study. The trend odds model is also presented in the application.

**Results**: Three types of models were fitted in one real‐life example, including ordered stereotype, proportional odds, and trend odds models. They reached similar conclusions in terms of the significance of covariates. The simulation study evaluated the performance of the ordered stereotype model under four cases. The performance varies depending on the scenarios.

**Conclusions**: The method presented can be applied to several areas of psychiatry dealing with ordinal outcomes. One of the main advantages of this model is that it breaks with the assumption of levels of the ordinal response are equally spaced, which might be not true.

## 1 INTRODUCTION

### 1.1 Background

An ordinal variable is one with a categorical data scale which describes order, and where the distinct levels of such a variable differ in degree of dissimilarity more than in quality (Agresti, 2010). In his seminal paper, Stevens (1946) called a scale ordinal if “any order‐preserving transformation will leave the scale form invariant” (p. 679). This article focuses on ordinal data which are very frequent in psychological and psychiatric studies where ordinal outcomes are often defined in several scales such as Likert scale (e.g., *strongly disagree*, *disagree*, *neither agree nor disagree*, *agree*, and *strongly agree*) and pain scale (e.g., from 0 to 10, where 0 means “no pain” and 10 means “extremely painful”). It is important to remark that the degree of dissimilarity among the adjacent levels of the scale in an ordinal variable might not necessarily be always the same. For instance, the difference in the severity of an injury expressed by level 2 rather than level 1 might be much more than the difference expressed by a rating of level 10 rather than 9.

Although the collection and use of ordinal variables is common, most of the current methods for analysing them treat the data as if they were continuous or nominal data (Hoffman & Franke, 1986). Agresti (2010, section 1.3) mentioned several disadvantages of using standard regression methods. First, the results are sensitive to the scores assigned. Second, it does not allow for the measurement that accounts for the error of replacing ordinal responses with continuous responses. Third, it can predict values outside the range of possible ordinal outcomes. Finally, another disadvantage of applying ordinary regression to ordinal data is to produce misleading results due to “floor” and “ceiling” effects on the dependent variable (see Agresti, 2010, section 1.3.1 and also comments regarding this issue in McKelvey & Zavoina, 1975; Winship & Mare, 1984; Bauer & Sterba, 2011; and Hedeker, 2015). Another common practice of dealing with ordinal outcomes is to dichotomize an ordinal variable with the aim of using logistic regression. However, Sanyeka and Weissfeld (1998) and Stromberg (1996) empirically showed that the effect estimates, precision, and predicting power could be very poor.

There are many existing methods developed for modeling ordinal data that respect the ordinal nature of the data and have advantages such as making as few assumptions as possible, having greater power for detecting relevant trends, and using measures that are similar to those used in ordinary regression for quantitative variables. Liu and Agresti (2005) and Agresti (2010) described various proportional odds version models using adjacent‐categories logits, cumulative logits (McCullagh, 1980), and continuation‐ratio logits (McCullagh & Nelder, 1989). In the literature, often, a proportional odds model refers to the one using cumulative logits, which is the most commonly used model for an ordinal response variable. The proportional odds structure makes a strong assumption on common odds ratios and this may be inadequate for some data. Alternatively, a partial proportional odds model by Peterson and Harrell (1990) allows non‐proportional odds for some or all covariates, but the model might contain many parameters, especially when there are many response categories. Recent research develop new methods to allow the flexibility on the proportional odds structure for modeling ordinal data such as the trend odds model (Capuano & Dawson, 2013; Capuano et al, 2016, Capuano, Wilson, Schneider, Leurgans, & Bennett, 2018) and the unconstrained and constrained versions of the partial adjacent category logit model (Fullerton & Xu, 2018). This article focuses on the ordered stereotype model introduced by Anderson (1984), which is also flexible compared with the model with the proportional odds structure as a result of adding additional score parameters. One of the main feature of this model is that it allows to determine a new spacing among the ordinal categories dictated by the data. The estimation of the spacing among ordinal responses is an improvement over other models for ordinal data.

The goal of this article is to introduce the ordered stereotype model to the researchers and practitioners in the field. We show its formulation, estimation, checking of overall fit, and its applications. Besides, we compare the ordered stereotype, proportional odds, and linear models. We use a simulation study to provide a guideline on the choice between these models.

This article is structured as follows. The data set used throughout this article is described in Section 1.2. Section 2 has definitions of the ordered stereotype model and provide various model checking tools. We illustrate the use of this model and evaluate the performance among the proportional odds, ordered stereotype, and ordinary linear models using a simulation study in Section 3. We conclude with a discussion, technical notes, and extensions in Section 4.

### 1.2 Data set

We use the data set from The Television School and Family Smoking Prevention and Cessation Project (TVSFP) study (Flay et al., 1988) throughout this article. This study was designed to test independent and combined effects of a school‐based social‐resistance curriculum and a television‐based program in terms of tobacco use prevention and cessation. One of the study outcomes is a tobacco and health knowledge (THKS) ordinal scale, which assesses the familiarity of students with tobacco and health. The sample consists of 1,600 7th‐grade students from 135 classrooms of 28 Los Angeles schools who had completed data on the THKS variable at both pretest and post‐test times. Table S1 in the Supplementary information summarizes the frequencies of THKS variable in an eight‐level ordinal scale. The most frequent categories are 1–4 (86.4% of the total), which present a similar frequency (between 18% and 25% of the total). From there, frequency in the first category is small and those from the last three categories decrease severely as the level of the ordinal response increases. The covariates were represented at Los Angeles school‐level. The 28 schools were randomized to either: (a) a social‐resistance classroom curriculum (CC), (b) a media (TV) intervention, (c) a combination of CC and TV, and (d) a no treatment control group. These conditions form a 2*x*2 design of CC (yes or no) by TV (yes or no). Table S2 in the Supplementary information describes all the variables and their possible values.

## 2 METHODS

### 2.1 The ordered stereotype model

Currently, the most frequently used in practice is probably the proportional odds model (Hosmer, Lemeshow, & Sturdivant, 2013, p. 297). It has the simplicity to interpret the covariate effect on ordinal responses due to the proportional odds assumption (McCullagh, 1980; Liu & Agresti, 2005; Agresti, 2010). Liu (2014) mentioned that because the proportional odds assumption is often violated, instead of using the partial proportional odds model (Peterson & Harrell, 1990), The stereotype model is an alternative option. It does not eliminate the other options, such as using the trend odd model (Capuano & Dawson, 2013, Capuano et al., 2016, 2018). Additionally, Greenland (1994) showed that the progression of a disease through various stages is naturally modeled by the stereotype model, and that the model is valid also under case dependent sampling, as opposed to the proportional odds model (Kuss, 2006). The stereotype model is not as popular as other equivalent ordinal regression models but it has been used in applied research (see e.g., Ananth & Kleinbaum, 1997 in epidemiology, Hendrickx & Gazenboom, 1998 in sociology, Guisan & Harrell, 2000 in ecology, and Lall, Campbell, Walters, & Morgan, 2002 and Abreu, Siqueira, Cardoso, & Caiaffa, 2008 in quality of life studies). Next, we give a general form of the model and discuss ways to check the overall quality of fit.

### 2.2 The ordered stereotype model. Formulation

Let *Y*_*i*_ be an ordinal response with *q* categories (e.g., strongly agree, agree, neutral, disagree, strongly disagree) for observation *i*, where *i*=1,…,*n*. The ordered stereotype model (Anderson, 1984) for the probability that *Y*_*i*_ takes the category *k* (*k*=1,…,*q*) is characterized by the following log odds:

(1) $$\begin{array}{cc} log\left(\frac{P\left[Y_{i}=k|x_{i}\right]}{P\left[Y_{i}=1|x_{i}\right]}\right) & =\alpha_{k}+\phi_{k}{\beta^{\prime }x}_{i},\;i=1,\text{⋯},n,\;k=2,\text{⋯},q, \end{array}$$

where the inclusion of the following monotone non‐decreasing constraint

(2) $$0=\phi_{1}\leq \phi_{2}\leq \text{⋯}\leq \phi_{q}=1$$

ensures that the response *Y*_*i*_ is ordinal (see Anderson, 1984). The vector ***x***_*i*_ is a set of predictor variables (covariates) for observation *i* which can be categorical or continuous, and the *p*×1 vector of parameters ***β*** represents the effects of ***x***_*i*_ on the log odds for the category *k*, relative to the baseline category of *Y*_*i*_. This formulation of the model treats the first category as the baseline category, the parameters {*α*_2_,…,*α*_*q*_} are the intercepts, and {*ϕ*_1_,*ϕ*_2_,…,*ϕ*_*q*_} are the parameters which can be interpreted as the “scores” for the categories of the response variable *Y*_*i*_. We restrict *α*_1_=*ϕ*_1_=0 and *ϕ*_*q*_=1 to ensure identifiability. With this construction, the response probabilities are as follows:

(3) $$\theta_{ik}=P\left[Y_{i}=k|x_{i}\right]=\frac{\exp(\alpha_{k}+\phi_{k}{\beta^{\prime }x}_{i})}{\sum_{\ell =1}^{q}\exp(\alpha_{\ell }+\phi_{\ell }{\beta^{\prime }x}_{i})}\;for\;k=1,\text{⋯},q.$$

An advantage of the stereotype model is that it is more parsimonious than the baseline category logit model that has the form $\alpha_{k}+{\beta_{k}^{\prime }x}_{i}$ on the right‐hand side of model (1). Additionally, the ordered stereotype model is more flexible than adjacent categories logits models with proportional odds structure (Agresti, 2010, section 4.3.4) as a result of the {*ϕ*_*k*_} parameters. Agresti (2010, see chapter 4) showed that the stereotype model is equivalent to the proportional odds version of the adjacent‐categories logit model, when the scores {*ϕ*_*k*_} are equally spaced. Although the model has advantages, it is not as popular as the proportional odds model, because the parameters are more difficult to estimate due to the intrinsic nonlinearity, which arises from the product of parameters in the predictor. However, the parameter estimates may be calculated by the standard maximum likelihood (ML) method (see, e.g., Agresti, 2010) by imposing the monotone nondecreasing constraint (2) through the reparametrization described in Fernández, Arnold, & Pledger (2016). To the best of our knowledge, there are a couple of fitting the stereotype model in **R** (R Core Team, 2013). The **R** packages for fitting the stereotype model in **R** (R Core Team, 2013). The **R** package ordinalgmifs (Archer et al., 2014) provides the function *ordinalgmifs* for fitting ordered stereotype models when the number of parameters exceeds the sample size, using the generalized monotone incremental forward stagewise method and imposing penalties to a set of chosen predictors. However, this package can be used also in the case of non‐high dimension data without specifying any penalty on the predictors in the model fitting process. This package includes a vignette (https://cran.r-project.org/web/packages/ordinalgmifs/vignettes/ordinalgmifs.pdf), which is a tutorial on fitting the ordinal stereotype model. Yee and Hastie (2003) fitted the stereotype model using the vector generalized additive model (VGAM) package (Yee, 2008), although it is not able to include the monotonic constraint in the score parameters. This paper obtains the maximum likelihood estimates of ordered stereotype models using the **R** function *ordinalgmifs*.

### 2.3 Estimation of non‐equal space among ordinal categories

**Figure 1:**
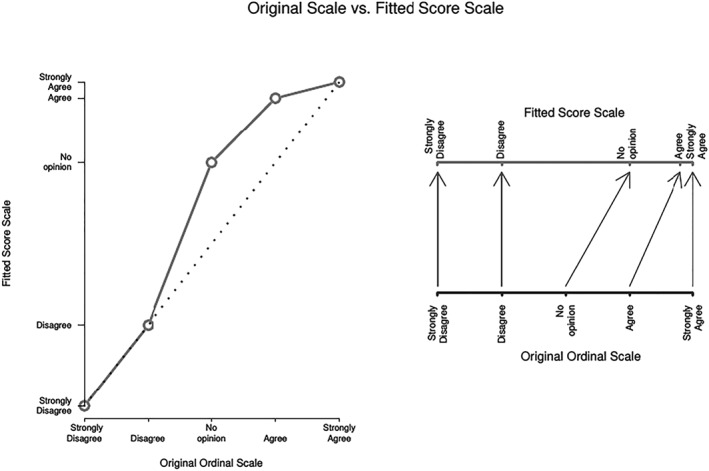
Reassigned ordinal scale: Scale comparison between default equal spacing and fitted spacing given by score parameters $\left\{{\hat{\phi }}_{k}\right\}$ for ordinal response variable with a 5‐level Likert scale (*strongly disagree*, *disagree*, *neither agree nor disagree*, *agree*, *strongly agree*)

An important remark regarding the use of ordinal responses is that the utilization of the first *q* positive integers as labels does not imply that there is an equal space among ordinal categories. The fitted spacing is instead determined by the distance among adjacent score parameters $\left\{{\hat{\phi }}_{k}\right\}$ and it could be different to the default equal spacing among its categories. To illustrate this, Figure 1 compares visually the default equal spacing with the fitted spacing. The data set for this example is related to the responses of 70 students giving feedback about a second year Applied Statistics course at Victoria University of Wellington. Model (1) was fitted with individual students and feedback questions as covariates. The figure depicts two graphs with a 5‐level Likert scale in an ordinal response variable (i.e., *strongly disagree*, *disagree*, *neither agree nor disagree*, *agree*, and *strongly agree*).

In the right graph, the equally spaced scale is depicted in the bottom axis and the fitted score scale is dictated by the data in the top axis. The fitted score parameters were ${\hat{\phi }}_{2}=0.252,{\hat{\phi }}_{3}=0.748,$ and ${\hat{\phi }}_{4}=0.946$ (*ϕ*_1_=0 and *ϕ*_5_=1 are restricted to ensure identifiability). The left graph shows a dotted straight line which corresponds to the equally spaced categories and the line depicts how different the fitted score parameters are from this uniformity. The amount of nonlinearity shows the distortion of the scale from the incorrect equally spaced scale. Therefore, the adjacent ordinal categories are not equally spaced based on the data.

We estimate the distance between two adjacent categories, for example, *k*+1 and *k*, based on *ϕ*_*k*+1_−*ϕ*_*k*_. For instance, the scores of ${\hat{\phi }}_{1}=0$, ${\hat{\phi }}_{2}=0.252$, ${\hat{\phi }}_{3}=0.748$, ${\hat{\phi }}_{4}=0.946$, and ${\hat{\phi }}_{5}=1$ imply that the spacing between categories *disagree* and *neither agree nor disagree* is the largest ( ${\hat{\phi }}_{3}-{\hat{\phi }}_{2}=0.496$) and the shortest ( ${\hat{\phi }}_{5}-{\hat{\phi }}_{4}=0.054$) is between *strongly agree* and *agree* categories. The categories *strongly agree* and *agree* are less distinguishable based on the information of individuals and feedback questions than the categories *disagree* and *neither agree nor disagree*. These two graphs allow us to easily depict the new uneven spacing of the levels of the ordinal response. Furthermore, if *ϕ*_*k*_=*ϕ*_*k*+1_, the covariates ***x*** do not distinguish between them. Therefore, we could collapse them as a single response category (Fernández et al., 2016; Agresti, 2010). To make the inference about how close these categories are, overlapping confidence intervals around the scores *ϕ*_*k*_ and *ϕ*_*k*+1_ may give evidence that ordinal categories *k* and *k*+1 are not distinguishable.

### 2.4 Check the model overall fit

One might express data in a contingency table where the columns are ordinal responses and the row variable represents all classifications/patterns for covariates. For instance, we can cross–classify all observations for the TVSFP data in Section 2 into a 4×8 contingency table with (a)–(d) groups (as described in Section 2) as rows and the eight ordinal scales as columns. The cell count for row *i* and column *j* is the number of subjects who were in the group *i* and responded on level *j*. The fitted cell counts are calculated from the estimated response probabilities (3).

In terms of assessing the adequacy of the fitted model for ordered stereotype models, the Pearson *X* ^2^ and the deviance *G* ^2^ statistics are two classical summary measures to compare the maximum likelihood fitted cell counts that satisfy Model (1) to the observed cell counts. They have the following forms (Agresti, 2007, pp. 35–36):

(4) $$\begin{array}{ccc} X^{2} & =\sum_{\text{all cells}}\frac{{\left(\text{observed cell count}-\text{fitted cell count}\right)}^{2}}{\text{fitted cell count}} & \\ G^{2} & =2\sum_{\text{all cells}}(\text{observed cell count})\log\left(\frac{\text{observed cell count}}{\text{fitted cell count}}\right). \end{array}$$

For the large sample theory, both test statistics follow an asymptotic chi–square distribution with *df*(number of logits from the left hand side of Model 1—number of parameters from the left hand side of Model 1) when almost all the fitted cell counts are at least 5. The asymptotic theory holds only when the model has covariates with few patterns. It does not hold when there is a continuous covariate. For the TVSFP data in Section 2, because the covariates result in four groups (a–d), there are (*q*−1)×4=(8−1)×4=28 logits from the left hand side of Model (1). The parameters for Model (1) include (*q*−1) of *α*_*k*_'s, (*q*−2) of *ϕ*_*k*_'s, and three group effects in *β*'s. Therefore, the test statistic has ((*q*−1)×4)−((*q*−1)+(*q*−2)+(4−1))=12 degrees of freedom.

When the large sample criterion does not hold for Pearson *X* ^2^ and the deviance *G* ^2^ statistics, Fernández and Liu (2016) proposed a goodness‐of‐fit test of the ordered stereotype model, $S_{g_{1},g_{2}}$. The test is based on the well‐known Hosmer–Lemeshow test (Hosmer & Lemeshow, 1980) and its version for the proportional odds regression model (Fagerland & Hosmer, 2013). The latter test statistic is calculated from a grouping scheme assuming that the levels of the ordinal response are equally spaced, which might not be true. The $S_{g_{1},g_{2}}$ test statistic takes the use of the new adjusted spacing to partition data as it uses the ordered stereotype model. Fernández and Liu (2016) showed the steps to construct the proposed test as follows:

- Calculate the estimated probabilities ${\hat{\theta }}_{ik}$ (Equation 3) for each observation *i*=1,…,*n* and response category *k*=1,…,*q*.
- Compute the weighted score for each observation:
  (5) $$s_{i}=\sum_{k=1}^{q}v_{k}\times {\hat{\theta }}_{ik},\;i=1,\text{⋯},n,$$
  where *v*_1_=1, *v*_*q*_=*q* and $v_{k}=1+(q-1)\times {\hat{\phi }}_{k}$. Note that the {*v*_*k*_} in the range of [1,*q*] are the rescaled ordinal scores for the response categories, calculated from the score parameter estimates $\left\{{\hat{\phi }}_{k}\right\}$ in [0,1].
- Replace the observed response {*y*_*i*_} for each observation by its corresponding rescaled ordinal scores {*v*_*k*_}, denoted by $\left\{{ŷ}_{i}\right\}$. For example, ${ŷ}_{i}=v_{k}$ if *y*_*i*_=*k*. Due to the nature of ordinal stereotype models, the spacing information between response categories is better captured by {*v*_*k*_}. As a result, the equal spacing between categories is removed by the new fitted spacing.
- Compute the deviances for each observation: $d_{i}=s_{i}-{ŷ}_{i}$ (*i*=1,…,*n*).
- Sort the *n* observations ascending by {*d*_*i*_}.
- Create a first partition into *g*_1_ groups of the data, such that each group *ℓ* contains *n*_*ℓ*_=*n*/*g*_1_ observations (*ℓ*=1,…,*g*_1_ and $n=n_{1}+n_{2}+\text{⋯}+n_{g_{1}}$). For instance, if *g*_1_=2, the data is divided into two portions in which each portion contains 50% of the observations. As a result of this step, the data are grouped according to the level of deviations. This is favorable to produce similar groups of observations based on their quality of fit (deviance). Fernández and Liu (2016) suggested to use *g*_1_=2.
- For each *g*_1_ group, we sort the corresponding {*n*_*ℓ*_,*ℓ*=1,…,*g*_1_} observations ascending by the weighted scores {*s*_*i*_}.
- For each *g*_1_ group, we create a second partition into *g*_2_ subgroups based on the weighted sorting scores {*s*_*i*_}, such that each subgroup contains {*n*_*ℓ*_/*g*_2_,*ℓ*=1,…,*g*_1_} observations.
- Cross classify the observations according to the *G*=*g*_1_×*g*_2_ groups and the ordinal response categories to create a *G*×*q* contingency table. The observed frequencies {*o*_*gk*_} and the estimated expected frequencies {*e*_*gk*_} under the model are defined as:
  $$\begin{array}{ccc} o_{gk} & =\sum_{\upsilon \in \Upsilon_{g}}I[y_{\upsilon }=k]\;\text{and}\;e_{gk}=\sum_{\upsilon \in \Upsilon_{g}}{\hat{\theta }}_{\upsilon k},\;\text{for} & \\ g & =1,\text{⋯},G,\;k=1,\text{⋯},q, \end{array}$$
  where Υ_*g*_ denotes the set of indices of the observations in group *g* and *I*[*A*] is a binary indicator that takes value 1 if *A* is true and 0 otherwise.
- Compute the Pearson *χ* ^2^ statistic $S_{g_{1},g_{2}}$ as:
  (6) $$S_{g_{1},g_{2}}=\sum_{g=1}^{G}\sum_{k=1}^{q}\frac{{\left(o_{gk}-e_{gk}\right)}^{2}}{e_{gk}},$$
  where *G*=*g*_1_×*g*_2_.

The $S_{g_{1},g_{2}}$ test statistic follows a *χ* ^2^ distribution with *df*=(*G*−2)(*q*−1)+(*q*−2) degrees of freedom when the fitted model is correct (see details in Fernández & Liu, 2016, section 3).

### 2.5 Check the ordinal assumption

Because the ordered stereotype model is a special case of the baseline–category logit model (also known as multinomial logistic regression)

(7) $$\begin{array}{cc} \log\left(\frac{P\left[Y_{i}=k|x_{i}\right]}{P\left[Y_{i}=1|x_{i}\right]}\right) & =\alpha_{k}+{\beta_{k}^{\prime }x}_{i}\;i=1,\text{⋯},n,\;k=2,\text{⋯},q, \end{array}$$

we could check the adequacy of the ordinal trend, that is, whether it is plausible to replace ${\beta_{k}^{\prime }x}_{i}$ by $\phi_{k}{\beta^{\prime }x}_{i}$ with 0=*ϕ*_1_ ≤ *ϕ*_2_ ≤ … ≤ *ϕ*_*q*_=1 using a likelihood ratio test. The test statistic has form:

(8) $$D=-2\log\left(\frac{\text{maximum likelihood for Model (1)}}{\text{maximum likelihood for Model (7)}}\right).$$

The test statistic follows an asymptotic *χ* ^2^ distribution with (*p*)×(*q*−1)−(*p*+(*q*−2))=*pq*−2*p*−*q*+2 degrees of freedom under the ordinal trend assumption. When there is only one covariate (*p*=1), the test statistic has zero degrees of freedom. The model fitting is the same between the baseline category logit model and the stereotype model without the monotone nondecreasing constraint (2). Therefore, the test is only valid for *p* ≥ 2.

Another possible model comparison test is to compare the proportional odds model with the ordered stereotype model. Given that the proportional odds model is more parsimonious than the ordered stereotype model, we also could check how much information has been missed by fitting a proportional odds model instead of an ordered stereotype model. As those two models are not nested, we could calculate an information criterion measure such as AIC and BIC to compare those models.

## 3 RESULT

### 3.1 Application

We fit the ordered stereotype model to the original eight‐level ordinal response THKS from the *n*=1,600 students using the covariates CC, TV, and their interaction CCTV. Note that we intentionally ignore the class and school levels here as we simply want to demonstrate the use of ordered stereotype model for independent observations. A two‐level mixed effects model allowing for nesting of students within classrooms can be applied allowing for nesting of students within classrooms using a Bayesian approach. We remark that we only used post‐test responses for simplicity. There are two ways to consider both pretest and post‐test responses. One is to treat the pretest response as a covariate. Another one is to include a subject‐specific random effect.

After model fitting, the estimates of the score parameters are ${\hat{\phi }}_{k}=(0,0.083,0.324,0.452,0.988,0.999,1,1)$, which shows an uneven spacing among ordinal outcomes. As we explained in Section 3.2, the closeness of the first two and last four score parameters implies that the set of covariates do not distinguish between those categories. We can therefore collapse those categories, and end up with only four ordinal categories. Table S3 in the Supplementary information summarizes the frequencies of the new four‐level variable (THKS4), which are now all quite balanced (between 22.2% and 27.9% of the total observations). The ordered stereotype model was fitted again using the same set of covariates and the response outcome THKS4.

Table 1 gives the result of the model fitting showing that the covariate social‐resistance classroom curriculum (CC) is significant at 0.05 level on the tobacco and health knowledge of the students. At 0.01 level, both covariates and their interaction have a significant effect on the response. The fitted scores shows uneven spacing ( $\{{\hat{\phi }}_{k}\}=(0,0.197,0.878,1),$ in which adjacent ordinal categories 3 and 4 are closer than 2 and 3, or 1 and 2.

**Table 1:** Results of fitting the ordered stereotype model (Equation 1) for the TVSFP data set. The four‐level response variable THKS4 is used

**Coefficient**	**Estimation**	**SE**	**95% CI**
${\hat{\alpha }}_{2}$	0.023	0.108	(−0.190,0.235)
${\hat{\alpha }}_{3}$	−0.341	0.126	(−0.587,−0.095)
${\hat{\alpha }}_{4}$	−0.305	0.133	(−0.565,−0.045)
${\hat{\beta }}_{1}$ (CC)	1.052***	0.202	(0.656,1.447)
${\hat{\beta }}_{2}$ (TV)	0.309*	0.169	(−0.021,0.639)
${\hat{\beta }}_{3}$ (CCTV)	−0.467*	0.252	(−0.962,0.027)
${\hat{\phi }}_{2}$	0.197	0.114	(0.083,0.311)
${\hat{\phi }}_{3}$	0.878	0.121	(0.757,0.999)

***Significant at .01 level.

**Significant at .05 level.

*Significant at .1 level.

Figure S1 in the Supplementary information illustrates how adjacent categories are not equally spaced for this data set. We might rescale $\left\{{\hat{\phi }}_{k}\right\}$ as ${\hat{\nu }}_{1}=1$, ${\hat{\nu }}_{q}=q$ and ${\hat{\nu }}_{k}=1+(q-1)\times {\hat{\phi }}_{k}$ in order to put the categories in its original range [1,*q*]. In this case, $\left\{{\hat{\nu }}_{k}\}=(1,1.59,3.63,4\right)$.

Regarding the goodness–of–fit of the model, it is important to remark that the test $S_{g_{1},g_{2}}$ might not fit well when all covariates are dichotomous variables because this produces a small number of covariates patterns and the approximate chi‐square distribution does not hold. Thus, as all covariates of the TVSFP study data set are dichotomous, we calculated both the Pearson *X* ^2^ and the deviance *G* ^2^ statistic tests for assessing the goodness‐of‐fit of the model, as discussed in Section 3.3. We calculated the 4×4 contingency table, which satisfies the requirement that all expected frequencies should be greater than 1 and at least 80% should be greater than 5 for a good $\chi_{\text{df}}^{2}$‐approximation. Table S4 in the Supplementary information gives the table of observed and expected frequencies by cross‐classifying the four collapsed ordinal response levels (columns) and the four covariate patterns (rows). The value of the tests are very similar (*X* ^2^=3.4299 and *G* ^2^=3.4297) giving the same *p* value < .489, which suggests no evidence of lack of fit at 5% of significance level.

We also calculated the AIC and BIC values to compare the baseline‐category logit model, the proportional odds model, and the ordered stereotype model for the TVSFP study data set. The results are shown in Table S5 in the Supplementary information. The ordered stereotype model is the best model according to AIC. However, the BIC values show that the proportional odds model is the best model, which makes sense because BIC penalizes less parsimonious models. Thus, there is not much information missed by fitting a proportional odds model instead of an ordered stereotype model for this data set. On the other hand, the baseline‐category logit model is the less appropriate model according to AIC and BIC, indicating that the ordinal assumption is necessary.

Finally, we fitted both the proportional odds and trend odds models to the application dataset (SAS script is available in the Supplementary information, Appendix 1 in Section B). The trend odds model assumes that the ordinal data are generated by a latent non‐standard logistic distribution, for example, logistic distribution with a scale parameter that is different from one, which makes the model more flexible in several cases. It assumes that nonproportional odds are monotonic so that a common slope (*γ*) could be used for different ordinal levels and requires to know the scaling between response categories (*t*_*k*_) in advance. For instance, Capuano and Dawson (2013) used *t*_*k*_=*k*−1. In contrast, spacing parameters (*ϕ*'s) in the ordered stereotype model are estimated from data. Table 2 shows the results for the comparison between the proportional odds and trend odds models for the TVSFP data set. The significant estimates of both models are similar. The discrepancy lies in the covariate CCTV, which is not significant in the trend odds model, but significant in the proportional odds model.

**Table 2:** Results of fitting the proportional odds model (POM) and the trend odds model (TOM) for the TVSFP data set. The four‐level response variable THKS4 is used

	**POM**		**TOM**	
**Coefficient**	**Estimation**	**SE**	**Estimation**	**SE**
${\hat{\alpha }}_{2}$	0.8890***	0.0937	0.8610***	0.0956
${\hat{\alpha }}_{3}$	−0.2752***	0.0906	−0.2730***	0.0897
${\hat{\alpha }}_{4}$	−1.3661***	0.0967	−1.3200***	0.1033
${\hat{\beta }}_{1}$ (CC)	0.7770***	0.1282	0.8158***	0.1630
${\hat{\beta }}_{2}$ (TV)	0.2244*	0.1239	0.2233***	0.0248
${\hat{\beta }}_{3}$ (CCTV)	−0.3720**	0.1799	−0.2743	0.2224
${\hat{\gamma }}_{1}$ (CC)	‐	‐	−0.0432	0.0862
${\hat{\gamma }}_{2}$ (TV)	‐	‐	−0.0022	0.0496
${\hat{\gamma }}_{3}$ (CCTV)	‐	‐	−0.0749	0.1026

Abbreviations: POM, proportional odds model; SE, standard error; TOM, trend odds model.

***Significant at .01 level.

**Significant at .05 level.

*Significant at .1 level.

**Figure 2:**
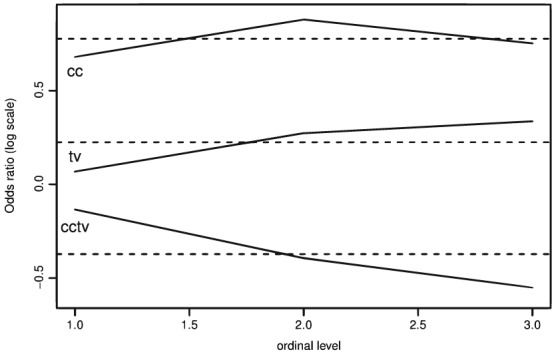
Graphical comparison between the proportional odds model and the nonproportional odds model: Ordinal response variable in the TVSFP study data set

Additionally, Figure 2 compares the proportional odds model and the nonproportional odds model. Using both likelihood ratio test *p* value = .2595) and score test *p* value = .2631), we conclude that the proportional odds model is adequate for the TVSFP study data set.

### 3.2 Simulation study

We set up a simulation study in a diverse range of scenarios with the aim of measuring how different the results are when the ordinality in the response variable is not taken into account properly using two cases. We also compare the choice of ordered stereotype and proportional odds models when neither of them is the true model in Case 3. In Case 4, in order to check the robustness of the ordered stereotype model, we compare the performance of the linear regression and ordered stereotype models when the true model is the linear regression model.

Case 1 The goal of Case 1 is to evaluate if we can keep the same set of predictors by naively treating the ordinal scales as equal space measurements to fit an ordinary linear regression model. On the basis of Agresti's findings (Agresti, 2010, section 1.3.1), the design of the models intentionally includes an interaction term between the covariates. We expect to have similar findings.

The data were generated from the following ordered stereotype model

(9) $$\begin{array}{ccc} \log\left(\frac{P\left[Y_{i}=k|x_{1},x_{2}\right]}{P\left[Y_{i}=1|x_{1},x_{2}\right]}\right) & =\alpha_{k}+\phi_{k}(\beta_{1}x_{i1}+\beta_{2}x_{i2}), & \\ i & =1,\text{⋯},n,\;k=2,\text{⋯},q, \end{array}$$

which does not include an interaction term between the covariates *x*_1_ and *x*_2_ and includes the monotone ordinal constraint (Equation 2) to ensure the ordinal nature of the data generated.

The fitted models include the linear regression model:

(10) $$E[Y_{i}|x_{1},x_{2}]=\alpha +\beta_{1}x_{i1}+\beta_{2}x_{i2}+\beta_{12}x_{i1}x_{i2},\;i=1,\text{⋯},n$$

and the ordered stereotype model as follows:

(11) $$\begin{array}{ccc} \log\left(\frac{P\left[Y_{i}=k|x_{1},x_{2}\right]}{P\left[Y_{i}=1|x_{1},x_{2}\right]}\right) & =\alpha_{k}+\phi_{k}(\beta_{1}x_{i1}+\beta_{2}x_{i2}+\beta_{12}x_{i1}x_{i2}), & \\ i & =1,\text{⋯},n,\;k=2,\text{⋯},q. \end{array}$$

We are interested in testing the hypothesis $H_{0}:\beta_{12}=0$ against $H_{1}:\beta_{12}\neq 0$ at a 5*%* significance level. Because the true model does not have the interaction effect, we should not reject the null hypothesis too often for both fitted models if we can keep the same set of predictors.

We simulated data from Equation (9) varying the response categories (*q*=3,4,5) and the covariate parameters (*β*_1_,*β*_2_). Table 3 shows a summary of the true parameters for the model, where the score parameters {*ϕ*_*k*_} were assigned to be equally spaced and the true parameters {*α*_*k*_} were chosen to avoid highly unbalanced frequencies in the response categories.

Two different scenarios were considered in regard with the distribution of the covariates *x*_1_ and *x*_2_. Scenario 1 has $x_{1}∼N(0,1)$ and *x*_2_∼Bern(0.5); and Scenario 2 has both *x*_1_ and *x*_2_ follow N(0,1) independently. For each case, we generated 5,000 data sets (replicates) of sample size *n*=500 and we calculated the proportion of times the hypothesis $H_{0}:\beta_{12}=0$ was rejected at a 5% level. Tables 4 and 5 show an overall summary of the results for different configurations of the covariate effect parameters (*β*_1_,*β*_2_) for Scenario 1 and Scenario 2 with *n*=500, respectively. The equivalent results for sample sizes *n*=100 and *n*=1,000 are shown in Tables S6–S9 in the Supplementary information.

The rejection rate of the test when an ordered stereotype model was fitted is close to the nominal level regardless different combinations of (*β*_1_,*β*_2_), which is expected. However, the results when a linear regression model was fitted are much worse, with rejection rates up to 29% (*q*=3, *β*_1_=1, *β*_2_=4 in Scenario 1). It confirms that no interaction term is no longer true by naively treating the ordinal scales as equal space measurements to fit an ordinary linear regression model. Additionally, Table 6 shows a summary table of the averages of all scenarios broken down by sample size. The stereotype model obtained the worst results for Scenario 1 when *n*=100, which makes sense. In that case, the values were a little bit higher than the 5% nominal level (6.26, 6.22, and 6.29 in average when *q*=3, 4, and 5, respectively), but the results are close to the 5% nominal level when the sample size increases. However, the linear regression model has an erratic behavior: it performs well when *n*=100 but when *n* increases, it performs badly. Moreover for Scenario 2 (i.e., two normal distributions), the ordered stereotype model performs well. It was quite the opposite for the linear regression model

Finally, we ran a sample of this simulation study but at 1% and 10% significance levels (not shown in this paper). The results were very similar to those at a 5% significance level.

It could be common to find unbalanced frequencies of the ordinal responses in data from real examples. In order to test that, we extended the scenarios in this case taking into account unbalanced ordinal frequencies. In particular, we ran simulations for the same Scenarios 1 and 2 and used different configurations of the covariate effect parameters (*β*_1_,*β*_2_). We modified the values of the intercepts {*α*} in order to get three types of unbalanced frequencies: (a) unbalanced towards lower ordinal categories (***α***=[0,0.2,−1.0,−1.6,−2.5]), (b) unbalanced towards mid ordinal categories (***α***=[0,0.2,1.0,−1.6,−2.5]), and (c) unbalanced towards higher ordinal categories (***α***=[0,−1.6,−2.5,0.2,1.0]). For each scenario, we generated 5,000 data sets (replicates) of sample size *n*=500 and *q*=5 and calculated the proportion of times the hypothesis $H_{0}:\beta_{12}=0$ was rejected at a 5% level. Table 7 gives the parameter setup and the results. It shows that the ordered stereotype model is robust to all unbalanced scenarios, whereas the linear regression model has a bad performance in all scenarios apart from some cases of the scenario where the unbalanced frequencies are towards mid ordinal categories.

**Table 3:** Parameters used to investigate the proportion of times that $H_{0}:\beta_{12}=0$ is rejected at a 5% significance level for the ordered stereotype model (Equation 9) for *q*=3,4,5 response categories

**Categories**		
**(** ***q*** **)**	**{*α*_*k*_}**	**{ *ϕ*_*k*_}**
**3**	(0,−0.6,−1.5)	(0,1/2,1)
**4**	(0,0.2,−0.8,−1.2)	(0,1/3,2/3,1)
**5**	(0,−0.1,−0.8,−1.2,−1.6)	(0,1/4,2/4,3/4,1)

**Table 4:** Proportion of times that $H_{0}:\beta_{12}=0$ was rejected at a 5% level with *n*=500, over 5,000 simulations for Scenario 1 ( $x_{1}∼N(0,1)$ and *x*_2_∼Bern(0.5)) when each of the LRM and the OSM was fitted

		***q*=3**		***q*=4**		***q*=5**	
***β*_1_**	***β*_2_**	**LRM**	**OSM**	**LRM**	**OSM**	**LRM**	**OSM**
0.50	2.5	6.82	4.36	5.53	5.50	4.90	5.07
0.75	2.5	8.42	4.14	5.54	5.42	5.16	5.04
1.00	2.5	10.31	4.38	5.18	5.32	4.98	5.82
0.50	3.0	8.51	4.93	5.78	4.83	7.28	4.68
0.75	3.0	12.34	4.26	6.85	4.92	6.84	4.46
1.00	3.0	15.54	4.18	7.20	4.79	7.82	5.10
0.50	3.5	10.24	5.12	6.08	4.97	8.78	4.98
0.75	3.5	16.02	4.18	9.04	4.82	8.48	4.52
1.00	3.5	21.55	5.15	10.92	5.18	10.83	4.72
0.50	4.0	11.12	4.85	7.62	5.15	10.31	5.28
0.75	4.0	21.68	5.04	11.42	5.18	12.95	4.77
1.00	4.0	29.35	4.29	14.21	4.98	13.91	5.02

Abbreviations: LRM, linear regression model; OSM, ordered stereotype model.

**Table 5:** Proportion of times that $H_{0}:\beta_{12}=0$ was rejected at a 5% level with *n*=500, over 5,000 simulations for Scenario 2 ( $x_{1}∼N(0,1)$ and $x_{2}∼N(0,1)$) when each of the LRM and the OSM was fitted

		***q*=3**		***q*=4**		***q*=5**	
***β*_1_**	***β*_2_**	LRM	OSM	LRM	OSM	LRM	OSM
1.0	2.5	10.18	5.14	7.52	5.98	10.18	6.34
2.0	2.5	23.36	4.52	14.44	6.12	19.52	6.14
3.0	2.5	26.46	4.54	18.41	5.48	23.56	5.54
1.0	3.0	9.62	5.12	6.56	5.30	8.14	6.06
2.0	3.0	23.06	4.54	15.58	5.24	20.62	6.22
3.0	3.0	28.86	4.68	19.86	4.96	24.72	5.72
1.0	3.5	8.14	4.78	6.22	5.30	9.16	5.94
2.0	3.5	21.66	4.25	14.66	5.68	19.52	5.74
3.0	3.5	27.94	5.17	20.16	5.08	26.61	5.44
1.0	4.0	6.94	4.24	5.62	4.94	6.84	5.56
2.0	4.0	18.16	4.46	13.84	4.78	16.32	4.67
3.0	4.0	26.82	5.13	19.74	4.24	25.7	4.32

Abbreviations: LRM, least regression model; OSM, ordered stereotype model.

**Table 6:** Proportion of times that $H_{0}:\beta_{12}=0$ was rejected at a 5% level, over 5,000 simulations when each of the LRM and the OSM was fitted, averaged over all the scenarios and broken down by sample size

		***q*=3**		***q*=4**		***q*=5**	
***Scenario***	***n***	LRM	OSM	LRM	OSM	LRM	OSM
1	100	5.43	6.26	5.36	6.22	5.54	6.29
	500	14.33	4.57	7.95	5.09	8.52	4.96
	1000	11.47	4.8	6.66	5.04	8.16	4.95
2	100	16.73	5.21	16.65	5.22	16.93	5.16
	500	19.27	4.71	13.55	5.26	17.57	5.64
	*1*000	16.77	5.21	19.56	5.14	16.85	5.2

Abbreviations: LRM, least regression model; OSM, ordered stereotype model.

**Table 7:** Proportion of times that $H_{0}:\beta_{12}=0$ was rejected at a 5% level with *n*=500 and *q*=5, over 5000 simulations for Scenario 1 ( $x_{1}∼N(0,1)$ and *x*_2_∼Bern(0.5)) and Scenario 2 ( $x_{1}∼N(0,1)$ and $x_{2}∼N(0,1)$) when each of the linear regression model (LRM) and the ordered stereotype model (OSM) was fitted. The values of the intercepts {*α*} are chosen to classify three types of unbalanced scenarios: a) towards lower ordinal categories (“Low”), b) towards mid ordinal categories (“Mid”), and c) towards higher ordinal categories (“High”)

			**Low**		**Mid**		**High**	
	***β*_1_**	***β*_2_**	**LRM**	**OSM**	**LRM**	**OSM**	**LRM**	**OSM**
**Scenario 1**	0.50	2.50	11.92	4.74	4.61	5.11	51.25	4.83
	0.75	3.00	20.68	5.00	4.65	5.00	88.95	5.30
	1.00	4.00	23.00	4.65	4.65	4.43	93.52	4.22
**Scenario 2**	0.50	2.50	20.21	5.01	4.28	5.12	13.24	5.63
	0.75	3.00	34.35	4.92	5.78	5.45	22.36	5.89
	1.00	4.00	48.98	4.47	10.28	5.28	31.05	5.62

Case 2 Consider three models as follows:

(12) $$\begin{array}{ccc} \log\left(\frac{P\left[Y_{i}=k|x_{1},\text{⋯},x_{p}\right]}{P\left[Y_{i}=1|x_{1},\text{⋯},x_{p}\right]}\right) & =\alpha_{k}+\phi_{k}(\beta_{1}x_{i1}+\text{⋯}+\beta_{p}x_{ip}), & \\ k & =2,\text{⋯},q, \end{array}$$

(13) $$\begin{array}{ccc} \log\left(\frac{P\left[Y_{i}\leq k|x_{1},\text{⋯},x_{p}\right]}{1-P\left[Y_{i}\leq k|x_{1},\text{⋯},x_{p}\right]}\right) & =\alpha_{k}+\beta_{1}x_{i1}+\text{⋯}+\beta_{p}x_{ip}, & \\ k & =1,\text{⋯},q-1, \end{array}$$

(14) $$E[Y_{i}|x_{1},\text{⋯},x_{p}]=\alpha +\beta_{1}x_{i1}+\text{⋯}+\beta_{p}x_{ip},$$

The goal of Case 2 is to evaluate main effects by comparing ordered stereotype (12), proportional odds (13), and ordinary linear (14) models. The true model includes relevant and noise covariates that allows us to check the size and power of a test for main effects. The data were generated from Model (12) or Model (13) under different scenarios listed in Table 8. The score parameters {*ϕ*_*k*_} ranges from equally spaced to highly unbalanced patterns and the true parameters {*μ*_*k*_} were chosen to avoid highly unbalanced frequencies in the response categories. The fitted models include all three models (12), (13), (14).

We are interested in testing the hypotheses $H_{0}:\beta_{1}=0$ against $H_{1}:\beta_{1}\neq 0$ and $H_{0}:\beta_{2}=0$ against $H_{1}:\beta_{2}\neq 0$ at a 5*%* significance level, respectively. For each scenario, we generated 5,000 data sets (replicates) of sample size *n*=500 and we calculated the proportion of times that the hypothesis $H_{0}:\beta_{h}=0$ was rejected at a 5% level for *h*=1, 2 using a likelihood ratio test statistic. When the true parameter equals 0, we obtain the size of a test. On the other hand, if the true parameter does not equal 0, the power of a test can be found. We set *β*_1_≠0 and *β*_2_=0 for all scenarios when there are two parameters (*p*=2) in a model to obtain both size and power of a test. Table 8 shows results for different configurations of the covariates *x*_1_ and *x*_2_ between N(5,3) and Bern(0.5) distributions.

When there is only one covariate, the performance of ordered stereotype models (12) is the best in terms of the size of tests, regardless the true model. The power of tests seems to be similar across three different fitted models. When there are two covariates, the performance of an ordered stereotype model (12) depends on the magnitude of the non–zero parameter. As the magnitude increases, the better the performance. Due to the multiplicative structure of *ϕ*_*k*_ and *β*'s, the performance of $\left\{{\hat{\phi }}_{k}\right\}$ relies on the non–zero *β*'s. Given a fixed sample size, $\left\{{\hat{\phi }}_{k}\right\}$ are further away from the true {*ϕ*_*k*_} if all *β*'s are closer to 0. That is, we cannot estimate the score parameters well if there is little information on covariates. It also applies to the cases when the non–zero *β* is associated with a binary covariate (e.g., S2211‐S2234). Besides, because of the multiplicative structure, for the scenarios with *p*=1, the likelihood ratio test statistic has an asymptotic chi‐square distribution with three degrees of freedom for an ordered stereotype model under $H_{0}$. The three degrees of freedom come from *β*, *ϕ*_2_, and *ϕ*_3_ under *q*=4.

The ordinary linear model (14) is the worst when the true score parameters are highly unbalanced (e.g., S2134). When data were generated from a proportional odds model (13), the result from fitting a linear model is not bad. The stereotype model fitting is also good, considering Scenario P2114 (with *β*_1_=1.00) only due to the multiplicative issue. When data were generated from a stereotype model with two continuous covariates, the proportional odds model fitting is slightly worse than the stereotype model fitting for a large *β*_1_ (=1.00).

From simulations in Cases 1 and 2, we conclude that when the predictor structure is complicated, that is, with interaction terms, results by fitting of a linear regression model are different from the true situation. For the cases with main effects only, fitting a linear model could also result in a misleading result when there are two or more covariates.

**Table 8:** True model columns show parameters used to generate data for *q*=4 response categories with *n*=500. Fitted model columns show proportions of times that $H_{0}:\beta_{h}=0$ was rejected at a 5% level, over 5,000 simulations with *h*=1, 2. When the true *β*_*h*_=0, the proportion = size of the test; and when the true *β*_*h*_≠0, the proportion = power of the test

	True Model							Fitted Model: Size/Power (in *%*)		
Scenario	Model	*p*	*x*_1_	*x*_2_	{*ϕ*_*k*_}	*β*_1_	*β*_2_	(12)	(13)	(14)
S1111	(12)	1	N(5,3)	‐	(0,1/3,2/3,1)	0	‐	4.94/‐	4.60/‐	4.62/‐
S1112	(12)	1	N(5,3)	‐	(0,1/3,2/3,1)	0.20	‐	‐/89.7	‐/86.4	‐/86.1
S2111	(12)	2	N(5,3)	N(5,3)	(0,1/3,2/3,1)	0.15	0	5.26/66.1	5.04/61.4	5.30/61.5
S2112	(12)	2	N(5,3)	N(5,3)	(0,1/3,2/3,1)	0.25	0	5.18/92.3	5.06/91.2	5.20/91.1
S2113	(12)	2	N(5,3)	N(5,3)	(0,1/3,2/3,1)	0.50	0	5.15/100	5.28/100	5.25/100
S2114	(12)	2	N(5,3)	N(5,3)	(0,1/3,2/3,1)	1.00	0	5.00/100	5.34/100	5.00/100
S2121	(12)	2	N(5,3)	N(5,3)	(0,0.2,0.8,1)	0.15	0	5.80/63.2	5.42/57.0	5.40/57.1
S2122	(12)	2	N(5,3)	N(5,3)	(0,0.2,0.8,1)	0.25	0	6.10/95.8	5.94/96.0	6.12/95.7
S2123	(12)	2	N(5,3)	N(5,3)	(0,0.2,0.8,1)	0.50	0	5.00/100	4.80/100	4.92/100
S2124	(12)	2	N(5,3)	N(5,3)	(0,0.2,0.8,1)	1.00	0	5.32/100	5.64/100	4.90/100
S2131	(12)	2	N(5,3)	N(5,3)	(0,0.3,0.998,1)	0.15	0	5.88/67.6	5.16/63.2	5.20/61.7
S2132	(12)	2	N(5,3)	N(5,3)	(0,0.3,0.998,1)	0.25	0	5.28/97.4	4.48/97.0	4.54/96.6
S2133	(12)	2	N(5,3)	N(5,3)	(0,0.3,0.998,1)	0.50	0	5.12/100	5.14/100	4.92/100
S2134	(12)	2	N(5,3)	N(5,3)	(0,0.3,0.998,1)	1.00	0	5.20/100	4.80/98.7	3.30/100
S2211	(12)	2	B(0.5)	N(5,3)	(0,1/3,2/3,1)	0.15	0	5.50/9.75	4.90/7.90	5.30/8.00
S2212	(12)	2	B(0.5)	N(5,3)	(0,1/3,2/3,1)	0.25	0	5.40/13.8	5.40/13.8	4.80/14.2
S2213	(12)	2	B(0.5)	N(5,3)	(0,1/3,2/3,1)	0.50	0	5.65/48.8	5.35/45.8	5.30/47.7
S2214	(12)	2	B(0.5)	N(5,3)	(0,1/3,2/3,1)	1.00	0	5.74/95.5	5.64/94.9	5.52/95.6
S2221	(12)	2	B(0.5)	N(5,3)	(0,0.2,0.8,1)	0.15	0	6.65/11.8	4.50/8.60	4.85/8.40
S2222	(12)	2	B(0.5)	N(5,3)	(0,0.2,0.8,1)	0.25	0	5.45/17.5	5.25/15.7	5.60/16.9
S2223	(12)	2	B(0.5)	N(5,3)	(0,0.2,0.8,1)	0.50	0	6.35/53.7	6.05/48.5	5.90/51.3
S2224	(12)	2	B(0.5)	N(5,3)	(0,0.2,0.8,1)	1.00	0	5.35/97.7	5.30/97.6	5.00/97.9
S2231	(12)	2	B(0.5)	N(5,3)	(0,0.3,0.998,1)	0.15	0	5.85/10.1	4.66/8.24	4.06/8.60
S2232	(12)	2	B(0.5)	N(5,3)	(0,0.3,0.998,1)	0.25	0	5.85/21.1	4.15/18.3	4.10/18.7
S2233	(12)	2	B(0.5)	N(5,3)	(0,0.3,0.998,1)	0.50	0	6.65/62.8	5.30/56.6	5.25/55.3
S2234	(12)	2	B(0.5)	N(5,3)	(0,0.3,0.998,1)	1.00	0	5.55/99.3	5.60/99.2	5.30/99.0
P1111	(13)	1	N(5,3)	‐	‐	0	‐	4.96/‐	4.64/‐	4.42/‐
P1112	(13)	1	N(5,3)	‐	‐	0.15	‐	‐/87.2	‐/89.2	‐/89.1
P2111	(13)	2	N(5,3)	N(5,3)	‐	0.15	0	5.88/85.6	4.84/84.3	4.56/81.5
P2112	(13)	2	N(5,3)	N(5,3)	‐	0.25	0	5.84/99.9	4.98/99.9	5.12/99.9
P2113	(13)	2	N(5,3)	N(5,3)	‐	0.50	0	5.56/100	5.12/100	5.22/100
P2114	(13)	2	N(5,3)	N(5,3)	‐	1.00	0	5.52/100	5.60/100	5.30/100

*Note*. The scenario is labeled by “Mabcd”, where M=S for Model (12) and M=P for Model (13); “a” indicates the number of covariates *p*; “b” indicates the distribution of *x*'s; “c” shows the structure of {*ϕ*_*k*_}; and “d” shows different values of *β*'s.

Case 3 When a baseline–categories logit model is the true model, the choice between ordered stereotype and proportional odds models might depend on the parameter structure in the baseline–categories logit model. We simulated several scenarios to investigate it.

The data were generated from the following baseline–categories logit model

(15) $$\begin{array}{ccc} \log\left(\frac{P\left[Y_{i}=k|x_{1},x_{2}\right]}{P\left[Y_{i}=1|x_{1},x_{2}\right]}\right) & =\alpha_{k}+\beta_{k1}x_{i1}+\beta_{k2}x_{i2}, & \\ i & =1,\text{⋯},n,\;k=2,\text{⋯},q, \end{array}$$

where *q*=4 and the true parameters {*α*_*k*_} were chosen to avoid highly unbalanced frequencies in the response categories. The covariates *x*_1_ and *x*_2_ were generated from N(5,3) with sample sizes *n*=100,500, and 1,000. If both {*β*_*k*1_} and {*β*_*k*2_} are monotonic increasing over *k*=1,…,*q*, it implies that the ordered stereotype model (1) would provide a good fit. The goal of the simulation study in Case 3 is to investigate the situations when it is not true.

We fitted both ordered stereotype (12) and proportional odds (13) models for each scenarios listed in Table 9. We compared the two fitted models using AIC. Table 9 shows the proportion of times over 5,000 simulations that the ordered stereotype model (12) has a lower AIC than the proportional odds model (13).

For Scenarios 1‐3, {*β*_*k*1_} are nondecreasing over *k*, but {*β*_*k*2_} may not have the same pattern. The ordered stereotype model (12) was preferable for these scenarios. However, when both {*β*_*k*1_} and {*β*_*k*2_} do not follow a monotonic increasing/decreasing pattern as in Scenario 4, the proportional odds model (13) was preferred, that is, we didn't gain much by adding additional parameters using ordered stereotype models in terms of the model fitting. Additionally, we also note that the larger the sample size, the larger the proportion of times that AIC results in favor of the stereotype model is. Moreover, those proportions converge to around 65% when *n*=1,000. Because there is generally a trade‐off between goodness‐of‐fit and parsimony, the choice of models depends on researcher's needs. If a better fit is not a big problem, the proportional odds model is more parsimonious and easier to interpret than the stereotype model.

**Table 9:** True model columns show parameters used in Model (15) to generate data for *q*=4 response categories with *n*=100,500, and 1,000. The last column gives the proportion of times that the ordered stereotype model (12) is better than the proportional odds model (13) over 5,000 simulations when the two models were fitted

Scenario	True model		AIC results in favor of (12) (in *%*)		
	{*β*_*k*1_}	{*β*_*k*2_}	*n*=100	*n*=500	*n*=1000
1	(0, 0.25, 0.50, 0.8)	(0, 0.5, −0.05, −0.5)	51.34	61.58	68.73
2	(0, 0.25, 0.50, 0.8)	(0, 0.5, −0.2, −0.5)	49.16	58.14	65.36
3	(0, 0.25, 0.50, 0.8)	(0, −0.2, −0.4, −0.5)	42.12	55.62	65.79
4	(0, 2.0, 2.1, 1.9)	(0, 0.5, −0.05, −0.5)	25.56	33.24	64.83

Case 4 With the aim of looking into robustness to misspecification of the ordered stereotype model, we set up a simulation study when the linear model is the true model. This case is similar to Case 1, but now the data was generated from Model (10) without the interaction effect under a diverse range of scenarios listed in the first two columns of Table 10. The fitted models are Models (10) and (11). We are interested in testing the same hypothesis about the interaction term between covariates *x*_1_ and *x*_2_: $H_{0}:\beta_{12}=0$ against $H_{1}:\beta_{12}\neq 0$ at a 5*%* significance level. Because the true model does not have the interaction effect, we should not reject the null hypothesis too often for both fitted models if we can keep the same set of predictors. Table 10 shows the results when $x_{1}∼N(0,1)$ and *x*_2_∼Bern(0.5) when *n*=500. The results for sample sizes *n*=100 and *n*=1000 are given in Tables S10 and S11 in the Supplementary information.

We can observe that the rejection rate of the test when the ordered stereotype model was fitted are very close to the 5% nominal level in all the scenarios, which shows that the model is quite adequate even though the true model is the linear regression model.

**Table 10:** Case 4. Proportion of times that $H_{0}:\beta_{12}=0$ was rejected at a 5% level with *n*=500, over 5,000 simulations for Scenario 1 ( $x_{1}∼N(0,1)$ and *x*_2_∼Bern(0.5)) when each of the LRM and the OSM was fitted

		***q*=3**		***q*=4**		***q*=5**	
***β*_1_**	***β*_2_**	LRM	OSM	LRM	OSM	LRM	OSM
0.50	2.5	3.98	4.12	5.20	5.50	4.98	5.08
0.75	2.5	5.06	4.97	4.83	4.60	4.22	3.98
1.00	2.5	5.07	4.74	4.92	5.06	4.80	4.80
0.50	3.0	5.12	4.67	4.58	4.61	4.92	5.18
0.75	3.0	4.91	5.00	5.58	5.52	4.79	5.28
1.00	3.0	5.03	5.01	4.77	4.96	4.79	5.66
0.50	3.5	5.15	4.65	5.00	5.33	5.04	5.28
0.75	3.5	5.08	4.76	5.30	4.80	5.04	5.29
1.00	3.5	5.01	5.02	5.00	5.00	4.95	5.30
0.50	4.0	4.84	4.72	4.78	4.12	4.69	4.55
0.75	4.0	4.70	4.62	4.68	4.76	4.55	4.48
1.00	4.0	5.13	4.67	4.84	4.68	4.69	4.75

Abbreviations: LRM, least regression model; OSM, ordered stereotype model.

## 4 DISCUSSION

Psychiatric studies often deal with ordinal outcomes. These variables do not follow a normal distribution and, therefore, the application of ordinary regression might produce misleading results due to, for instance, “floor” and “ceiling” effects. This article has introduced a regression model developed for the analysis of ordinal data, the ordered stereotype model. Its use has several benefits such as making as few assumptions as possible, having greater power for detecting relevant trends, and using measures that are similar to those used in ordinary regression for quantitative variables (Agresti, 2010, section 1.2). One of the main advantages of this model is that it breaks with the assumption of levels of the ordinal response are equally spaced, which might be not true. We particularly focused on this model because it is straightforward to obtain score parameter estimates to determine a new uneven spacing of the ordinal outcomes.

The application of this model to different ordinal data structures, which are common in many psychiatric research studies, has been demonstrated. For independent observations, the formulation of the model, estimation of its parameters, and assessment of the adequacy of the fitted model have been presented. This paper also discusses the problem of treating ordinal responses as continuous using a simulation study. One might lead to a misleading result by fitting an ordinary linear regression model if there is more than one covariate. The simulation study also compare the differences between proportional odds and ordered stereotype models. When the true ordered stereotype model has equally spaced scores, fitting a proportional odds model seems plausible. However, it gets worse when the score parameters are highly unbalanced.

The use of the models and methods described in this article may be advantageous for practitioners in the field. Assigning nonequal scores to ordinal categories gives an easy way to show the spacing among ordinal categories. If practitioners have some knowledge about the score for each of the ordered categories, assigning scores might be the best way to analyse data, because ordinary linear models can be applied. However, if practitioners do not have any predetermined idea about the spacing between adjacent categories, the use of an ordered stereotype model is convenient as the data dictate the nonequally spaced scores among ordinal outcomes. Thus, for independent observations, descriptive statistics can be calculated using the new scores of ordinal scales. It may benefit the practitioners who can easily understand the mean or median as summary statistics.

This article has attempted to present the models and its application in the less technical possible way. The program for checking the ordered stereotype model overall fit was written in **R**. Meanwhile, the code is available upon request to the authors.

The estimation of the spacing among ordinal responses is an improvement over other ordinal data models such as proportional odds model and continuation‐ratio model, although more research in performance comparison with others equivalent methods is needed. Additionally, the development of methods for multilevel ordinal data (clustered and longitudinal data) where the ordered stereotype model were the underlying model might be a field to explore for future research.

## Supporting information

Supporting info item
