# Supplementary material for: A method for ordinal outcomes: The ordered stereotype model
Source: Int J Methods Psychiatr Res. 2019 Sep 30;28(4):e1801. doi: 10.1002/mpr.1801 (PMC7027430; doi:10.1002/mpr.1801)
Supplement: Supplementary file 1 — Supporting info item [file MPR-28-e1801-s001.pdf]

Supplementary Material for "A Method for Ordinal Outcomes: The Ordered Stereotype Model"

Daniel Fernández[1], Ivy Liu[2], and Roy Costilla[3]

1. Fundació Sant Joan de Déu; 2. Victoria University of Wellington; 3. University of Queensland

Author Note

Daniel Fernández, Research and Development Unit, Parc Sanitari Sant Joan de Déu, Fundació Sant Joan de Déu, CIBERSAM.

Ivy Liu, School of Mathematics and Statistics, Victoria University of Wellington.

Roy Costilla, Institute for Molecular Bioscience, University of Queensland, Brisbane, Australia.

Correspondence concerning this article should be addressed to Daniel Fernández, Research and Development Unit, Parc Sanitari Sant Joan de Déu, Dr. Antoni Pujadas, 42, Sant Boi de Llobregat, Barcelona 08830, Spain. E-mail: df.martinez@pssjd.org

Acknowledgments: The authors are sincerely grateful to Prof. Brian Flay for permission to use the TVSFP data set, and Prof. Donald Hedeker for providing the data set.

Supplementary Material for "A Method for Ordinal Outcomes: The Ordered Stereotype Model"

A. Television School and Family Smoking Prevention and Cessation Project (TVSFP) data set

Table 1 summarizes the frequencies of THKS variable in an 8-level ordinal scale and Table 2 describes all the variables and their possible values.

B. Application

For the TVSFP study, Table 3 summarizes the frequencies of the new 4-level variable (THKS4) and Figure 1 illustrates how adjacent categories are not equally spaced. The AIC and BIC values of the baseline-category logit model, the proportional odds model, and the ordered stereotype model for the TVSFP study data set are shown in Table 5. Table 4 shows the table of observed and expected frequencies by cross-classifying the four collapsed ordinal response levels (columns) and the four covariate patterns (rows). **The SAS script to fit the trend odds model for the application dataset is given in Appendix 1.**

C. Simulation Study

Tables 6-9 give the results for $n = 100$ and $n = 1000$ respectively for the two scenarios of Case 1. Tables 10 and 11 shows the results for Case 4.

Appendix 1: The SAS script to fit the trend odds model for the application dataset

```
FILENAME REFFILE 'TVSFP_v2.csv';


PROC IMPORT DATAFILE=REFFILE
DBMS=CSV
OUT=tvsfp;
GETNAMES=YES;
RUN;


PROC CONTENTS DATA=tvsfp; RUN;


**********STEP 2 **********;
***** POM *****;
proc logistic data=tvsfp desc;
model thkso= cc tv cctv;
run;
/* Score Test for the Proportional Odds Assumption */
/* Chi-Square DF Pr > ChiSq */
/* 7.6730 6 0.2631 */
/* cc   1 0.7770 0.1282 36.7184 <.0001 */
/* tv   1 0.2244 0.1239 3.2817 0.0701 */
/* cctv   1 -0.3720 0.1799 4.2735 0.0387 */


**********STEP 3 **********;
***** TOM cc *****;
proc nlmixed data=tvsfp;
parms Int1 = -2.5793 Int2 = -1.7325 Int3 = -0.8301 beta_cc -0.5 trend_cc = 0;
p1= 1/(1 + exp(-(Int1 + trend_cc*2*cc + beta_cc * cc )));
```

```
p2= 1/(1 + exp(-(Int2 + trend_cc*1*cc + beta_cc * cc)));
p3= 1/(1 + exp(-(Int3 + trend_cc*0*cc + beta_cc * cc)));
if thkso =4 then p = p1;
else if thkso =3 then p = p2-p1;
else if thkso =2 then p = p3-p2;
else p = 1-p3;
p = (p>0 and p<=1)*p + (p<=0)*1e-8 + (p>1);
loglik = log(p);
model thkso ~ general(loglik);run;
/* beta_cc 0.6837 0.1192 1600 5.73 <.0001 0.4498 0.9176 -0.00129 */
/* trend_cc -0.08322 0.06965 1600 -1.19 0.2323 -0.2198 0.05340 -0.00120 */

***** TOM tv *****;
proc nlmixed data=tvsfp;
parms Int1 = -2.5793 Int2 = -1.7325 Int3 = -0.8301 beta_tv -0.5 trend_tv = 0;
p1= 1/(1 + exp(-(Int1 + trend_tv*2*tv + beta_tv * tv )));
p2= 1/(1 + exp(-(Int2 + trend_tv*1*tv + beta_tv * tv)));
p3= 1/(1 + exp(-(Int3 + trend_tv*0*tv + beta_tv * tv)));
if thkso =4 then p = p1;
else if thkso =3 then p = p2-p1;
else if thkso =2 then p = p3-p2;
else p = 1-p3;
p = (p>0 and p<=1)*p + (p<=0)*1e-8 + (p>1);
loglik = log(p);
model thkso ~ general(loglik);run;
/* beta_tv 0.04578 0.1151 1600 0.40 0.6908 -0.1799 0.2715 -0.00004 */
/* trend_tv 0.01031 0.06691 1600 0.15 0.8775 -0.1209 0.1415 -0.00014 */

***** TOM cctv *****;
```

```
proc nlmixed data=tvsfp;
parms Int1 = -2.5793 Int2 = -1.7325 Int3 = -0.8301 beta_cctv 0.2 trend_cctv = 0.2;
p1= 1/(1 + exp(-(Int1 + trend_cctv*2*cctv + beta_cctv * cctv )));
p2= 1/(1 + exp(-(Int2 + trend_cctv*1*cctv + beta_cctv * cctv)));
p3= 1/(1 + exp(-(Int3 + trend_cctv*0*cctv + beta_cctv * cctv)));
if thkso =4 then p = p1;
else if thkso =3 then p = p2-p1;
else if thkso =2 then p = p3-p2;
else p = 1-p3;
p = (p>0 and p<=1)*p + (p<=0)*1e-8 + (p>1);
loglik = log(p);
model thkso ~ general(loglik);run;
/* beta_cctv 0.4637 0.1445 1600 3.21 0.0014 0.1803 0.7471 0.000676 */
/* trend_cctv -0.1317 0.08259 1600 -1.59 0.1110 -0.2937 0.03029 0.001440 */

***** TOM cc + tv + cctv *****;
proc nlmixed data=tvsfp;
parms Int1 = -2.5793 Int2 = -1.7325 Int3 = -0.8301
beta_cc 0.7770 trend_cc = 0
beta_tv 0.2244 trend_tv = 0
beta_cctv -0.3720 trend_cctv = 0;
p1= 1/(1 + exp(-(Int1 + trend_cc*2*cc + beta_cc*cc + trend_tv*2*tv + beta_tv*tv +
    trend_cctv*2*cctv + beta_cctv*cctv)));
p2= 1/(1 + exp(-(Int2 + trend_cc*1*cc + beta_cc*cc + trend_tv*2*tv + beta_tv*tv +
    trend_cctv*1*cctv + beta_cctv*cctv)));
p3= 1/(1 + exp(-(Int3 + trend_cc*0*cc + beta_cc*cc + trend_tv*2*tv + beta_tv*tv +
    trend_cctv*0*cctv + beta_cctv*cctv)));
if thkso =4 then p = p1;
else if thkso =3 then p = p2-p1;
```

```
else if thkso =2 then p = p3-p2;

else p = 1-p3;

p = (p>0 and p<=1)*p + (p<=0)*1e-8 + (p>1);

loglik = log(p);

model thkso ~ general(loglik);run;


/* Parameter Estimate Standard */

/* Error DF t Value Pr > |t| 95% Confidence Limits Gradient */

/* Int1 -1.3202 0.1033 1600 -12.77 <.0001 -1.5229 -1.1175 0.000375 */

/* Int2 -0.2729 0.08971 1600 -3.04 0.0024 -0.4489 -0.09696 -0.00019 */

/* Int3 0.8608 0.09559 1600 9.01 <.0001 0.6733 1.0483 -0.00038 */

/* beta_cc 0.8158 0.1630 1600 5.01 <.0001 0.4961 1.1354 -5.02E-6 */

/* trend_cc -0.04322 0.08616 1600 -0.50 0.6160 -0.2122 0.1258 0.000131 */

/* beta_tv 0.2233 0.02479 1600 9.01 <.0001 0.1747 0.2719 0.000241 */

/* trend_tv -0.00215 0.04958 1600 -0.04 0.9654 -0.09940 0.09509 0.000482 */

/* beta_cctv -0.2743 0.2224 1600 -1.23 0.2177 -0.7106 0.1620 0.000100 */

/* trend_cctv -0.07485 0.1026 1600 -0.73 0.4657 -0.2761 0.1264 0.000400 */
```

Table 1

Frequencies of the THKS variable, in an 8-level ordinal scale of the TVSFP study.

**THKS score**	**0**	**1**	**2**	**3**	**4**	**5**	**6**	**7**	**Total**
Frequency	67	288	398	400	296	119	28	4	1600
Percentage	4.2	18.0	24.9	25.0	18.5	7.4	1.8	0.2	

Table 2

Variables and their possible values of the TVSFP study.

Type	Variable	Description	Values	Freq. (%)
Response	THKS	Tobacco and health knowledge	Ordinal scale from 0-7 From less to more knowledge	
Covariates	CC	Social-resistance classroom curriculum	0 = No	52.3
			1 = Yes	47.7
	TV	Media intervention	0 = No	50.1
			1 = Yes	49.9
	CCTV	CC by TV interaction	0 = No	76.1
			1 = Yes	23.9

Table 3

Frequencies of the THKS4 variable, in a new 4-level ordinal scale.

**THKS4 score**	**1**	**2**	**3**	**4**	**Total**
Frequency	355	398	400	447	1600
Percentage	22.2	24.9	25.0	27.9	

Table 4

TVSFP study data set*: Observed and expected frequencies summed into the* 4×4 *contingency table in which all expected frequencies are greater than 1 and at least 80% are greater than 5.*

**Group**	**THKS4 = 1**		**THKS4 = 2**		**THKS4 = 3**		**THKS4 = 4**		**Total**
	**Obs.**	**Exp.**	**Obs.**	**Exp.**	**Obs.**	**Exp.**	**Obs.**	**Exp.**	**(n = 1600)**
(a) a social-resistance classroom (CC=1, TV=0)	62	61.7	78	77.7	106	110.4	134	130.2	380
(b) a media intervention (CC=0, TV=1)	110	103.4	105	112.4	91	96.4	110	103.8	416
(c) a combination of (a) and (b) (CC=1, TV=1)	66	68.6	86	83.8	114	106.9	117	123.6	383
(d) a no-treatment group (CC=0, TV=0)	117	121.3	129	124.1	89	86.2	86	89.4	421

Table 5

Model comparison. The minimum values for each information criterion are indicated in boldface

Model	AIC	BIC
Ordered Stereotype Model	**4389.7**	4432.7
Proportional Odds Model	4390.0	**4422.3**
Baseline-Category Logit Model	4394.2	4458.8

Table 6

Case1. Proportion of times that $\mathcal{H}_0 : \beta_{12} = 0$ was rejected at a 5% level with $n = 100$, over 5000 simulations for Scenario 1 ($x_1 \sim \mathcal{N}(0, 1)$ and $x_2 \sim$ Bern(0.5)) when each of the linear regression model (LRM) and the ordered stereotype model (OSM) was fitted.

β_1	β_2	$q = 3$		$q = 4$		$q = 5$	
		**LRM**	**OSM**	**LRM**	**OSM**	**LRM**	**OSM**
0.50	*2.5*	3.88	6.68	4.06	6.74	4.12	6.72
0.75	*2.5*	4.16	6.85	4.12	6.02	4.34	6.04
1.00	*2.5*	4.28	6.74	4.15	5.74	4.66	6.84
0.50	*3.0*	5.84	7.02	4.96	7.02	5.78	7.14
0.75	*3.0*	5.47	7.06	5.88	7.18	5.88	7.03
1.00	*3.0*	6.12	6.64	5.78	6.28	5.76	6.63
0.50	*3.5*	4.82	5.84	5.03	5.9	5.88	5.76
0.75	*3.5*	4.38	6.07	4.94	5.82	4.96	5.28
1.00	*3.5*	6.12	5.48	4.86	5.78	4.44	5.86
0.50	*4.0*	6.35	5.38	6.94	6.19	6.84	6.02
0.75	*4.0*	6.68	6.01	6.77	6.02	6.9	6.11
1.00	*4.0*	7.04	5.38	6.88	5.98	6.91	6.06

Table 7

Case1. Proportion of times that $\mathcal{H}_0 : \beta_{12} = 0$ was rejected at a 5% level with $n = 1000$, over 5000 simulations for Scenario 1 ($x_1 \sim \mathcal{N}(0,1)$ and $x_2 \sim$ Bern(0.5)) when each of the linear regression model (LRM) and the ordered stereotype model (OSM) was fitted.

β_1	β_2	$q = 3$		$q = 4$		$q = 5$	
		**LRM**	**OSM**	**LRM**	**OSM**	**LRM**	**OSM**
0.50	*2.5*	6.12	4.78	6.38	4.64	7.26	4.67
0.75	*2.5*	6.28	4.67	6.34	4,87	8.88	4.32
1.00	*2.5*	8.87	4.32	8.90	4.44	12.05	4.22
0.50	*3.0*	6.08	4.96	6.12	5.03	9.66	5.64
0.75	*3.0*	6.21	4.74	5.06	4.86	10.12	5.36
1.00	*3.0*	8.28	4.69	5.78	4.88	11.46	5.34
0.50	*3.5*	6.12	5.04	6.12	5.12	6.12	5.00
0.75	*3.5*	10.18	4.37	6.96	4.96	6.14	4.78
1.00	*3.5*	12.23	5.25	5.86	5.01	5.64	4.95
0.50	*4.0*	8.12	5.12	6.74	5.12	8.12	5.02
0.75	*4.0*	26.13	4.70	7.62	5.66	6.12	5.12
1.00	*4.0*	32.97	5.01	8.00	5.72	6.34	4.98

Table 8

Case1. Proportion of times that $\mathcal{H}_0 : \beta_{12} = 0$ was rejected at a 5% level with $n = 100$, over 5000 simulations for Scenario 2 ($x_1 \sim \mathcal{N}(0,1)$ and $x_2 \sim \mathcal{N}(0,1)$) when each of the linear regression model (LRM) and the ordered stereotype model (OSM) was fitted.

β_1	β_2	$q = 3$		$q = 4$		$q = 5$	
		**LRM**	**OSM**	**LRM**	**OSM**	**LRM**	**OSM**
1.0	*2.5*	9.26	5.91	9.31	5.91	9.42	5.76
2.0	*2.5*	18.50	5.46	18.89	5.75	18.59	5.65
3.0	*2.5*	22.53	5.22	22.09	5.19	23.06	5.14
1.0	*3.0*	8.29	5.48	7.91	5.42	7.98	5.44
2.0	*3.0*	20.47	5.22	19.08	5.46	19.77	5.22
3.0	*3.0*	23.72	5.20	24.7	5.12	24.81	5.02
1.0	*3.5*	7.97	5.40	7.79	5.24	8.07	5.24
2.0	*3.5*	18.18	5.33	18.61	5.34	18.48	5.16
3.0	*3.5*	25.65	5.23	24.35	5.22	25.37	5.2
1.0	*4.0*	6.50	4.83	6.60	4.85	6.39	4.81
2.0	*4.0*	16.27	4.66	16.3	4.64	16.48	4.66
3.0	*4.0*	23.40	4.60	24.15	4.53	24.68	4.59

Table 9

Case1. Proportion of times that $\mathcal{H}_0 : \beta_{12} = 0$ was rejected at a 5% level with $n = 1000$, over 5000 simulations for Scenario 2 ($x_1 \sim \mathcal{N}(0,1)$ and $x_2 \sim \mathcal{N}(0,1)$) when each of the linear regression model (LRM) and the ordered stereotype model (OSM) was fitted.

β_1	β_2	$q=3$		$q=4$		$q=5$	
		**LRM**	**OSM**	**LRM**	**OSM**	**LRM**	**OSM**
1.0	*2.5*	9.34	5.82	9.21	5.85	9.37	5.82
2.0	*2.5*	18.84	5.6	19.15	5.54	19.27	5.64
3.0	*2.5*	22.69	5.16	23.01	5.19	22.96	5.2
1.0	*3.0*	8.03	5.48	8.05	5.51	8.16	5.47
2.0	*3.0*	19.61	5.32	19.64	5.36	19.89	5.30
3.0	*3.0*	24.59	5.14	24.66	5.10	24.32	5.10
1.0	*3.5*	7.86	5.36	7.89	5.31	7.76	5.32
2.0	*3.5*	18.71	5.22	18.71	5.23	18.62	5.22
3.0	*3.5*	24.85	5.24	46.75	4.88	24.92	5.23
1.0	*4.0*	6.51	4.95	6.49	4.95	6.44	4.95
2.0	*4.0*	16.01	4.63	16.17	4.63	16.22	4.64
3.0	*4.0*	24.15	4.55	35.03	4.12	24.30	4.55

Table 10

Case4. Proportion of times that $\mathcal{H}_0 : \beta_{12} = 0$ was rejected at a 5% level with $n = 100$, over 5000 simulations for Scenario 1 ($x_1 \sim \mathcal{N}(0,1)$ and $x_2 \sim$ Bern(0.5)) when each of the linear regression model (LRM) and the ordered stereotype model (OSM) was fitted.

β_1	β_2	$q = 3$		$q = 4$		$q = 5$	
		**LRM**	**OSM**	**LRM**	**OSM**	**LRM**	**OSM**
0.50	*2.5*	3.89	4.12	4.22	5.62	4.88	5.22
0.75	*2.5*	5.28	5.18	5.12	5.7	4.69	4.18
1.00	*2.5*	4.82	5.07	4.91	4.78	5.05	5.83
0.50	*3.0*	4.96	4.70	4.60	5.20	4.71	5.21
0.75	*3.0*	4.81	5.14	4	6.4	4.92	4.64
1.00	*3.0*	4.89	5.15	4.58	5.64	4.71	5.51
0.50	*3.5*	4.92	4.88	3.88	6.36	4.40	4.54
0.75	*3.5*	4.87	5.06	4.46	6.47	4.70	4.29
1.00	*3.5*	5.17	5.05	4.74	5.12	4.98	5.60
0.50	*4.0*	4.68	5.03	4.26	5.50	4.41	5.42
0.75	*4.0*	4.99	4.63	3.68	7.02	4.76	5.40
1.00	*4.0*	4.75	5.14	5.00	4.80	4.94	4.92

Table 11

Case4. Proportion of times that $\mathcal{H}_0 : \beta_{12} = 0$ was rejected at a 5% level with $n = 1000$, over 5000 simulations for Scenario 1 ($x_1 \sim \mathcal{N}(0,1)$ and $x_2 \sim$ Bern(0.5)) when each of the linear regression model (LRM) and the ordered stereotype model (OSM) was fitted.

β_1	β_2	$q = 3$		$q = 4$		$q = 5$	
		**LRM**	**OSM**	**LRM**	**OSM**	**LRM**	**OSM**
0.50	*2.5*	4.12	4.26	3.80	4.40	5.07	5.03
0.75	*2.5*	5.18	4.64	4.00	5.66	4.09	5.33
1.00	*2.5*	5.10	4.60	4.89	4.86	4.29	4.67
0.50	*3.0*	4.86	5.19	4.76	4.66	4.76	5.02
0.75	*3.0*	4.85	5.20	4.14	3.98	4.18	4.83
1.00	*3.0*	4.94	4.75	4.88	5.76	4.79	4.06
0.50	*3.5*	4.96	4.71	5.00	5.50	4.42	4.56
0.75	*3.5*	4.99	4.60	4.66	4.52	5.46	4.26
1.00	*3.5*	4.90	4.74	4.78	4.88	4.07	4.37
0.50	*4.0*	4.95	5.12	4.89	4.89	4.04	5.04
0.75	*4.0*	5.03	4.71	4.81	5.12	4.83	5.28
1.00	*4.0*	5.17	4.74	4.70	5.10	4.80	5.63

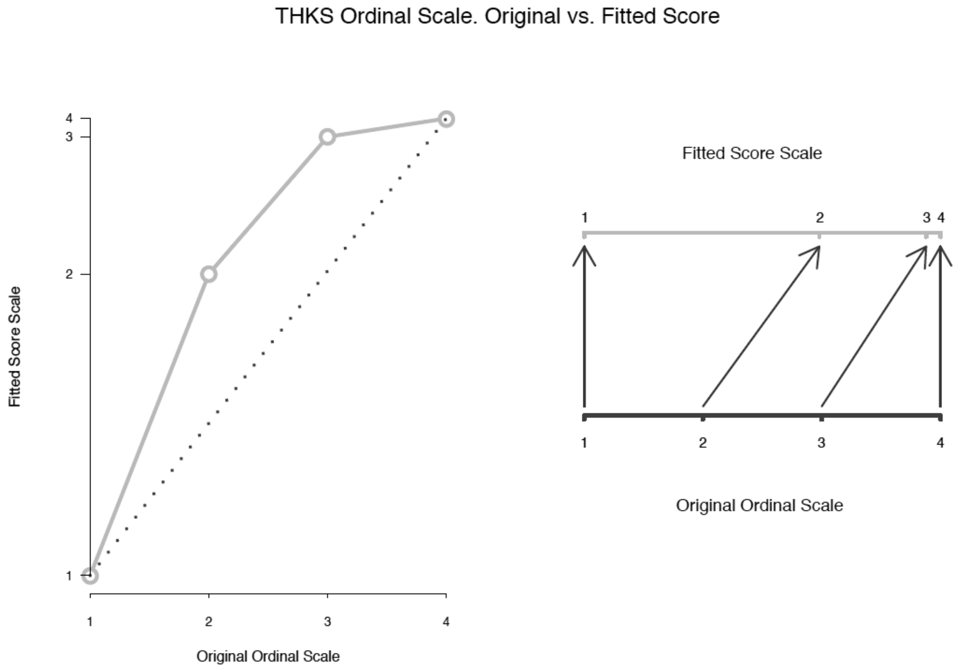

Figure 1. **Reassigned ordinal scale:** Scale comparison between default equal spacing and fitted spacing given by score parameters $\{\widehat{\phi}_k\}$ for ordinal response variable in the TVSFP study data set.
